# Trends in Use of Telehealth Among Health Centers During the COVID-19 Pandemic — United States, June 26–November 6, 2020

**DOI:** 10.15585/mmwr.mm7007a3

**Published:** 2021-02-19

**Authors:** Hanna B. Demeke, Sharifa Merali, Suzanne Marks, Leah Zilversmit Pao, Lisa Romero, Paramjit Sandhu, Hollie Clark, Alexey Clara, Kendra B. McDow, Erica Tindall, Stephanie Campbell, Joshua Bolton, Xuan Le, Julia L. Skapik, Isaac Nwaise, Michelle A. Rose, Frank V. Strona, Christina Nelson, Charlene Siza

**Affiliations:** ^1^CDC COVID-19 Response Team; ^2^Health Resources and Services Administration, U.S. Department of Health and Human Services, Rockville, Maryland; ^3^National Association of Community Health Centers, Bethesda, Maryland.

Telehealth can facilitate access to care, reduce risk for transmission of SARS-CoV-2 (the virus that causes coronavirus disease 2019 [COVID-19]), conserve scarce medical supplies, and reduce strain on health care capacity and facilities while supporting continuity of care. Health Resources and Services Administration (HRSA)–funded health centers* expanded telehealth^†^ services during the COVID-19 pandemic (*1*). The Centers for Medicare & Medicaid Services eliminated geographic restrictions and enhanced reimbursement so that telehealth services–enabled health centers could expand telehealth services and continue providing care during the pandemic (*2*,*3*). CDC and HRSA analyzed data from 245 health centers that completed a voluntary weekly HRSA Health Center COVID-19 Survey^§^ for 20 consecutive weeks to describe trends in telehealth use. During the weeks ending June 26–November 6, 2020, the overall percentage of weekly health care visits conducted via telehealth (telehealth visits) decreased by 25%, from 35.8% during the week ending June 26 to 26.9% for the week ending November 6, averaging 30.2% over the study period. Weekly telehealth visits declined when COVID-19 cases were decreasing and plateaued as cases were increasing. Health centers in the South and in rural areas consistently reported the lowest average percentage of weekly telehealth visits over the 20 weeks, compared with health centers in other regions and urban areas. As the COVID-19 pandemic continues, maintaining and expanding telehealth services will be critical to ensuring access to care while limiting exposure to SARS-CoV-2.

In April 2020, HRSA began administering a voluntary weekly Health Center COVID-19 Survey to track the effect of COVID-19 on health centers’ testing capacity, operations, patients, and staff members. Potential respondents were 1,382 HRSA-funded health centers. CDC and HRSA analyzed data from 245 health centers that responded to the Health Center COVID-19 Survey for 20 consecutive weeks (weeks ending June 26–November 6, 2020) to examine trends in telehealth use, assess differences by U.S. Census region^¶^ and urbanicity,** and compare telehealth patterns with the 7-day average number of new known COVID-19 cases within the counties where the included health centers were located. Region and urbanicity have previously been shown to be strongly associated with telehealth use (*1*). Each health center recorded the percentage of weekly telehealth visits in intervals of five (range = 0–100). Compared with health centers that responded at least once to the Health Center COVID-19 Survey over the study period (range = 912–1,011), consecutively responding health centers were more often located in more urban geographic areas (62.9% versus 58.5%) and in the Northeast (20.4% versus 16.7%) and West (29.4% versus 27.1%) regions than were those that did not report consecutively.

The average percentage of weekly telehealth visits was calculated nationally by region, and by urbanicity. Overall and weekly changes in telehealth visits (absolute percentage point difference and percentage change) for the first 10 weeks (weeks ending June 26–August 28, 2020) and the second 10 weeks (weeks ending September 4–November 6, 2020) were calculated. Weekly changes were calculated as the average of week-to-week differences in weekly telehealth visits for the first and second 10 weeks of the study period. The Kruskal-Wallis test was used to measure differences in the overall average percentage of weekly telehealth visits by region and urbanicity. Post hoc tests for pairwise comparisons were conducted if the Kruskal-Wallis test identified a significant main effect. P-values <0.05 were considered statistically significant. The 7-day average number of new known COVID-19 cases from 210 counties where the 245 health centers were located was accessed from USAFacts^††^ and calculated for each week of the survey and compared with the average percentage of weekly telehealth visits nationally by region and urbanicity. SPSS Statistics software (version 20; IBM) was used to conduct all analyses. These activities were reviewed by CDC and were conducted consistent with applicable federal law and CDC policy.^§§^

The overall average percentage of weekly telehealth visits among 245 consecutively responding health centers decreased 25%, from 35.8% during the week ending June 26, to 26.9% for the week ending November 6, averaging 30.2% over the study period (Table). Health centers in the South census regions and rural areas reported the lowest average percentage of weekly telehealth visits compared with health centers in other census regions and urban areas. During the first 10 weeks of study (June 26–August 28), health centers in the Northeast reported the largest absolute change in average percentage of weekly telehealth visits (–13.3%) followed by health centers in the Midwest (–7.3%). Urban health centers reported a larger absolute change in percentage of weekly telehealth visits for both the first 10 weeks (–7.6%) and the second 10 weeks of study (–3.0) compared with the change in rural health centers (–2.6% and –1.1%, respectively). Health centers in the Northeast reported stable weekly telehealth visits, with an absolute change of 0.1% over the second 10 weeks of study (September 4–November 6). Within each region and urbanicity stratum, the overall change in average percentage of telehealth visits differed significantly between the first 10 weeks and the last 10 weeks of the study period.

**TABLE:** Percentage of weekly telehealth visits* among consecutively responding Health Resources and Services Administration (HRSA)-funded health centers^†^ (N = 245), by U.S. Census region^§^ and urbanicity — Health Center COVID-19 Survey, United States, June 26–November 6, 2020

Regions	Total no. of health centers (%)	Weekly telehealth visits, week ending, no. (%)				Change in average percentage of weekly telehealth visits, absolute difference^¶^ (%)			
		Overall**	Jun 26	Aug 28	Nov 6	Overall,** Jun 26–Aug 28	Weekly,^††^ Jun 26–Aug 28	Overall,** Sept 4–Nov 6	Weekly,^††^ Sept 4– Nov 6
**U.S. Census region**									
Northeast	**50.0 (20.4)**	37.7 (0–100)^§§^	48.3 (0–95)	35.0 (0–80)	35.1 (0–95)	−13.3 (−27.5)	−1.5 (−3.5)	0.1 (0.3)	0.01 (0.2)
Midwest	**41.0 (16.7)**	28.4 (0–90)	36.0 (0–90)	28.7 (0–80)	24.5 (0–80)	−7.3 (−20.3)	−0.8 (−2.4)	−3.3 (−11.8)	−0.4 (−1.5)
South	**77.0 (31.4)**	20.4 (0–95)^§§^	22.7 (0–95)	21.6 (0–90)	17.8 (0–90)	−1.1 (−4.9)	−0.1 (−0.5)	−2.7 (−13.0)	−0.4 (−1.9)
West	**72.0 (29.4)**	36.3 (0–100)^§§^	41.0 (0–90)	36.3 (0–85)	32.3 (0–85)	−4.7 (−11.5)	−0.5 (−1.3)	−3.0 (−8.5)	−0.4 (−1.0)
Puerto Rico^¶¶^	**5.0 (2.0)**	31.3 (0–80)	35.0 (5–65)	32.0 (15–65)	28.0 (5–70)	−3.0 (−8.6)	−0.3 (0.8)	−1.0 (−3.4)	−0.4 (−0.5)
**Urbanicity**									
Urban	**154.0 (62.9)**	35.2 (0–100)^§§^	42.0 (0–95)	34.4 (0–90)	30.9 (0–95)	−7.6 (−18.1)	−0.8 (−2.2)	−3.0 (−8.7)	−0.4 (−1.0)
Rural	**91.0 (37.1)**	21.7 (0–95)	25.2 (0–90)	22.6 (0–90)	20.1 (0–90)	−2.6 (−10.2)	−0.3 (−1.1)	−1.1 (−5.2)	−0.3 (−1.1)
**Total**	**245.0 (100)**	**30.2 (0–100)^§§^**	**35.8 (0–95)**	**30.1 (0–90)**	**26.9 (0–95)**	−**5.7 (−16.0)**	−**0.6 (−1.9)**	−**2.3 (−7.8)**	−**0.3 (−1.1)**

**Abbreviation:** COVID-19 = coronavirus disease 2019.

* Percentage of weekly visits conducted virtually.

^†^ Health centers include HRSA-funded Federally Qualified Health Centers, which fall under the Consolidated Health Center Program (Section 1905(l)(2)(B) of the Social Security Act). Only data from HRSA-funded Federally Qualified Health Centers are included in this analysis.

^§^ *Northeast*: Connecticut, Maine, Massachusetts, New Hampshire, New Jersey, New York, Pennsylvania, Rhode Island, and Vermont; *Midwest*: Illinois, Indiana, Iowa, Kansas, Michigan, Minnesota, Missouri, Nebraska, North Dakota, Ohio, South Dakota, and Wisconsin; *South*: Alabama, Arkansas, Delaware, District of Columbia, Florida, Georgia, Kentucky, Louisiana, Maryland, Mississippi, North Carolina, Oklahoma, South Carolina, Tennessee, Texas, Virginia, and West Virginia; *West*: Alaska, Arizona, California, Colorado, Hawaii, Idaho, Montana, Nevada, New Mexico, Oregon, Utah, Washington, and Wyoming.

^¶^ Change calculated as absolute difference in percentage points.

** Results are based on data for the entire study period (weeks ending June 26–November 6, 2020).

^††^ Average of week-to-week differences in the average percentage of weekly visits conducted by telehealth, for the study period (weeks ending June 26–November 6, 2020).

^§§^ Kruskal-Wallis Test shows significant differences in the overall average percentage of telehealth visits by U.S. Census regions (p<0.01). Health centers in the South had lower overall average percentages of telehealth visits compared with those in the Northeast (p<0.01) and the West (p<0.01). Urban health centers had higher overall average percentages of telehealth visits than did rural health centers (p<0.01).

^¶¶^ Consecutively responding health centers in dependent areas for the study period included only those in Puerto Rico.

The overall average percentage of weekly telehealth visits differed significantly among some regions. Pairwise comparisons found that the overall average percentage in the South was significantly lower than that in the Northeast (p<0.01) and the West (p<0.01). The percentages of telehealth visits in the Northeast and the West did not differ significantly (p = 0.793). Urban health centers reported a significantly higher overall average percentage of telehealth visits than did rural health centers (p<0.01). The number of COVID-19 cases varied by region and increased overall during the second half of the study period (Figure 1). The increase in COVID-19 cases in the Northeast in the second 10 weeks of the study period aligned with the plateauing and slightly increasing trend in average percentage of weekly telehealth visits. The number of COVID-19 cases in the counties where urban health centers were located peaked in the week ending July 4 in the first 10 weeks and consistently increased in the second 10 weeks (Figure 2). However, the average percentage of weekly telehealth visits continued to trend downward.

**FIGURE 1:**
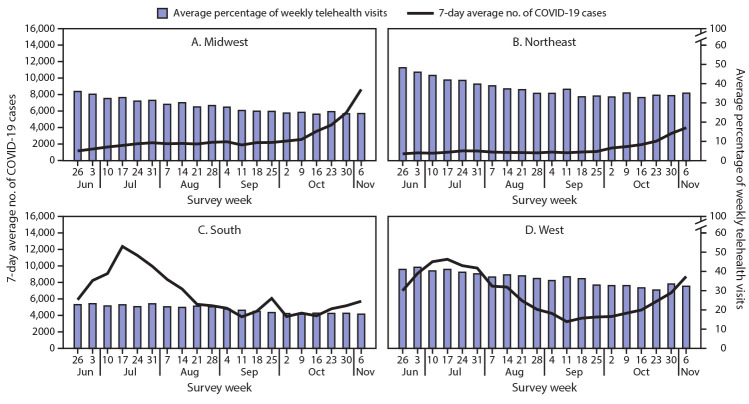
Average percentage of weekly telehealth visits* among consecutively responding^†^ Health Resources and Services Administration (HRSA)–funded health centers^§^ (N = 245) and 7-day average number of incident COVID-19 cases,^¶^ by U.S. Census region** **—** United States June 26–November 6, 2020 **Abbreviation:** COVID-19 = coronavirus disease 2019. * Percentage of weekly visits conducted virtually. ^†^ Health centers that responded to the voluntary weekly HRSA Health Center COVID-19 Survey each week for 20 weeks. ^§^ Health centers include HRSA-funded Federally Qualified Health Centers, which fall under the Consolidated Health Center Program (Section 1905(l)(2)(B) of the Social Security Act). Only data from HRSA-funded Federally Qualified Health Centers are included in this analysis. **^¶^** Seven-day average number of incident COVID-19 cases was calculated for each week of the study period for the 210 counties where 245 consecutively responding health centers are located. ** Dependent areas are not included because of the low number (five) reporting from this region.

**FIGURE 2:**
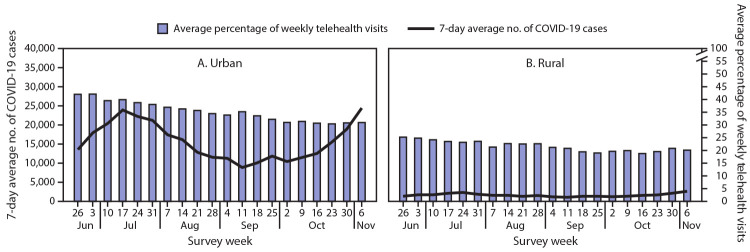
Average percentage of weekly telehealth visits* among consecutively responding^†^ Health Resources and Services Administration (HRSA)–funded health centers^§^ (N = 245) and 7-day average number of incident COVID-19 cases,^¶^ by urbanicity** **—** United States, June 26–November 6, 2020 **Abbreviation:** COVID-19 = coronavirus disease 2019. * Percentage of weekly visits conducted virtually. ^†^ Health centers that responded to the voluntary weekly HRSA Health Center COVID-19 Survey each week for 20 weeks. ^§^ Health centers include HRSA-funded Federally Qualified Health Centers, which fall under the Consolidated Health Center Program (Section 1905(l)(2)(B) of the Social Security Act). Only data from HRSA-funded Federally Qualified Health Centers are included in this analysis. ^¶^ Seven-day average number of COVID-19 cases was calculated for each week of the study period for the 210 counties where 245 consecutively responding health centers are located. ** Data presented do not include health centers in U.S. dependent areas because daily COVID-19 county-level case data were not available from USAFacts (https://usafacts.org/).

## Discussion

Health centers have expanded telehealth visits considerably; nearly one third of health visits were conducted using telehealth during the study period. According to 2019 Health Center Program Data,^¶¶^ 43% of health centers were capable of providing telemedicine, compared with 95% of the health centers that reported using telehealth during the COVID-19 pandemic (*1*). The largest increase in use of telehealth was reported in April 2020 (*4*,*5*). Following the release of Guidelines for Opening Up America Again*** on April 16, health care facilities resumed in-person visits. As COVID-19 cases declined from April to June, in-person care increased, and telehealth visits decreased (*4*,*5*). During June through late July, telehealth visits continued to decline, but at a slower rate in the South, where the number of COVID-19 cases sharply increased. Weekly telehealth visits plateaued beginning in mid-September, concomitant with another national surge of COVID-19 cases. Although in-person visits are needed to provide timely routine care and for urgent and emergency situations, maintaining telehealth capacity is critical during the COVID-19 pandemic. Telehealth visits can facilitate patient triage, which can reduce the effect of patient surge on facilities, address limitations to health care access, conserve personal protective equipment, and reduce disease transmission (*6*).

Health centers in the South and in rural areas have disproportionately experienced challenges and barriers, including the logistics of implementing telehealth, lack of partners or providers, and limited broadband access (*7*). State policies to provide financial assistance for telehealth infrastructure and technical guidance to providers facilitate telehealth implementation in underserved areas (*8*). Policy and practice changes under the COVID-19 Public Health Emergency proclamation (*2*) have enabled health centers to augment telehealth through the issuance of federal guidance and the subsequent support of federal resources. However, these additional resources might have a limited effect on barriers affecting patients, who need reliable broadband and communication devices capable of supporting telehealth as well as support on how to effectively use technology for telehealth visits (*7*,*9*). Programs that provide access to compatible devices and incorporate technical assistance to patients for virtual care to ensure productive encounters can reduce barriers to receipt of quality telehealth services. Assessment of disparities in access to and use of telehealth across population subgroups will be important in the future.

The findings in this report are subject to at least three limitations. First, the analysis was limited to health centers that consecutively reported data to HRSA during the study period and might not be representative of all health centers. Second, the analysis was limited to unweighted averages of percentages of weekly telehealth visits because numbers of telehealth visits are not recorded in the Health Center COVID-19 Survey. Finally, the number of COVID-19 cases in counties where health centers are located might not fully reflect the effect of the COVID-19 community transmission on the health center’s provision of telehealth visits.

Although resumption of in-person health care visits is anticipated, ongoing community transmission of SARS-CoV-2 might delay the transition to prepandemic levels of in-person care. Telehealth is critical to improving access to health care, especially among populations with limited access to care, and to enhancing the U.S. health care system’s capacity to continue to respond to the pandemic. HRSA-funded health centers have played a critical role as primary care providers by providing testing, treatment, and preventive care, including vaccination (*10*). As the COVID-19 pandemic continues, provision and expansion of health services using telehealth is critical to maintaining access to care while limiting exposure to SARS-CoV-2. Sustaining expanded use of telehealth visits in health centers during and after the pandemic might require continuation of existing flexibilities provided under Centers for Medicare & Medicaid Services telehealth reimbursement policies (*2*,*3*) and local level considerations of additional support and resources.

**Summary**

**What is already known about this topic?**

Telehealth can facilitate access to care, reduce risk for transmission of SARS-CoV-2, conserve scarce medical supplies, and reduce strain on health care capacity and facilities while supporting continuity of care.

**What is added by this report?**

During June 26–November 6, 2020, 30.2% of weekly health center visits occurred via telehealth. Telehealth visits declined as the number of new COVID-19 cases decreased but plateaued as the number of cases increased. Health centers in the South and rural areas consistently reported the lowest average percentage of weekly telehealth visits.

**What are the implications for public health practice?**

As the COVID-19 pandemic continues, maintaining the expansion of telehealth remains critical to providing access to care.

## Footnotes

* Data from HRSA-funded health centers (i.e., Federally Qualified Health Centers) are included for the analysis presented.

† Telehealth consists of the use of electronic information and telecommunication technologies to support clinical health care, patient and professional health-related education, public health, and health administration. https://www.hrsa.gov/rural-health/telehealth

§
https://bphc.hrsa.gov/emergency-response/coronavirus-health-center-data/

¶ *Northeast*: Connecticut, Maine, Massachusetts, New Hampshire, New Jersey, New York, Pennsylvania, Rhode Island, and Vermont; *Midwest*: Illinois, Indiana, Iowa, Kansas, Michigan, Minnesota, Missouri, Nebraska, North Dakota, Ohio, South Dakota, and Wisconsin; *South*: Alabama, Arkansas, Delaware, District of Columbia, Florida, Georgia, Kentucky, Louisiana, Maryland, Mississippi, North Carolina, Oklahoma, South Carolina, Tennessee, Texas, Virginia, and West Virginia; *West*: Alaska, Arizona, California, Colorado, Hawaii, Idaho, Montana, Nevada, New Mexico, Oregon, Utah, Washington, and Wyoming; *U.S. territories*: American Samoa, Puerto Rico, and U.S. Virgin Islands; *Freely associated states*: Federated States of Micronesia, Marshall Islands, and Palau. https://www.census.gov/geographies/reference-maps/2010/geo/2010-census-regions-and-divisions-of-the-united-states.html

** Urban/rural classification is based on the Federal Office of Rural Health Policy criteria. https://www.hrsa.gov/rural-health/about-us/definition/index.html

†† Daily COVID-19 case counts were obtained from USAFacts. https://usafacts.org/articles/detailed-methodology-covid-19-data/

§§ 45 C.F.R. part 46, 21 C.F.R. part 56; 42 U.S.C. Sect.241(d); 5 U.S.C. Sect.552a; 44 U.S.C. Sect.3501 et seq.

¶¶
https://data.hrsa.gov/tools/data-reporting/program-data/national/table?tableName=ODE&year=2019

***
https://assets.documentcloud.org/documents/6840714/Guidelines.pdf
